# Comparison of Health Literacy on Physical Activity and Nutrition Between Children and Adolescents with Congenital Heart Disease and Healthy Controls

**DOI:** 10.3390/jcdd13020058

**Published:** 2026-01-23

**Authors:** Isabel Uphoff, Charlotte Schöneburg, Renate Oberhoffer-Fritz, Peter Ewert, Jan Müller

**Affiliations:** 1 Department of Congenital Heart Disease and Pediatric Cardiology, Deutsches Herzzentrum München, Technische Universität München, 80636 München, Germany; 2 Institute of Preventive Pediatrics, Technische Universität München, 80636 München, Germany

**Keywords:** congenital heart disease, health literacy, physical activity, nutrition

## Abstract

Background: Health literacy can be defined as the ability to access, understand, evaluate, and apply health information in order to make informed health decisions in daily life. Despite its importance, little is known about health literacy in the domains of physical activity and nutrition among children with congenital heart disease. The aim of this study was to examine differences in health literacy related to physical activity and nutrition between children and adolescents with congenital heart disease and a healthy control group (Control). Methods: A total of 244 children and adolescents (age 8–18 years; mean age 13.4 ± 3.1 years, 53.3% girls) were enrolled, comprising 122 patients with congenital heart disease and 122 age- and sex-matched healthy controls. Patients were recruited during routine medical examinations at the German Heart Center, while controls were recruited from Munich schools. The Physical Activity Health Literacy Scale for Children (PAHL-C) and the Nutrition Health Literacy Scale for Children (NHL-C) were used to assess health literacy in the domains of physical activity and nutrition. Scores for both scales were calculated as additive sum scores and converted to a scale from 0 to 100, where higher scores indicate better health literacy. Physical activity levels were measured via self-reported frequency of days per week in which children engaged in at least one hour of physical activity. Results: Children with congenital heart disease showed lower PAHL-C scores (Patients: 65.9 ± 18.0 vs. Control: 72.9 ± 14.9, *p* = 0.001) and lower NHL-C scores (Patients: 63.6 ± 19.0 vs. Control: 69.3 ± 14.8, *p* = 0.009) than healthy controls. Boys reported significantly higher self-reported physical activity levels (*p* = 0.001) and had significantly higher PAHL-C scores than girls (*p* < 0.001). Patients with congenital heart disease reported significantly less physical activity compared to controls (Patients: 4.2 ± 1.7 days/week vs. Control: 5.2 ± 1.8 days/week, *p* < 0.001). Conclusions: Children with congenital heart disease demonstrate lower health literacy in the domains of physical activity and nutrition than their healthy counterparts and report being less active. This highlights the need for targeted interventions to address both physical activity and health literacy in children with congenital heart disease.

## 1. Introduction

Children with congenital heart defects represent a growing population group. Worldwide, more than 4.18 million children are affected by congenital heart disease, representing a 3.4% increase since 1990 [1]. Moreover, advances in medical treatment have significantly increased survival rates, and 97% of children with congenital heart disease reach adulthood [2]. Therefore, lifestyle factors such as physical activity and nutrition are becoming increasingly important for maintaining health and improving quality of life. A healthy lifestyle contributes to increased life expectancy and overall well-being, whereas unhealthy behaviors are associated with a higher risk of developing comorbidities [3,4,5].

Health literacy can be defined as the “knowledge, motivation and competences to access, understand, appraise, and apply health information in order to make judgments and take decisions in everyday life concerning healthcare, disease prevention and health promotion to maintain or improve quality of life during the life course” [6]. The World Health Organization (WHO) further identifies health literacy as an important social determinant of health [7]. There is broad consensus among researchers and policymakers that promoting health literacy from an early age is crucial for self-determination, long-term health and maintaining a good quality of life throughout life [8]. Higher levels of health literacy have been associated with healthier lifestyle behaviors, including higher levels of physical activity [9,10].

Previous research has demonstrated that higher health literacy in the congenital heart disease population group is associated with better overall health outcomes, improved treatment adherence and self-management, whereas low health literacy is linked to poorer outcomes, increased complications, and higher healthcare costs [11]. However, it remains unclear to what extent this population possesses health literacy specifically related to physical activity and nutrition.

At the same time, children with congenital heart disease tend to be less physically active than their peers [12]. Brudy et al. found that especially children with congenital heart disease who are overweight or obese, have complex heart defects, or have total cavopulmonary connection did not meet the WHO criteria for 60 min of moderate to vigorous physical activity per day [13]. They also face numerous barriers to engaging in physical activity, such as individual (e.g., anxiety and low self-efficacy), sociocultural (e.g., lack of knowledge, parental fear), and environmental barriers (e.g., limited access) [14]. Therefore, the present study aims to investigate whether differences exist in health literacy on physical activity and nutrition between children with congenital heart disease and healthy controls.

## 2. Materials and Methods

### 2.1. Study Participants

This cross-sectional study enrolled 122 children and adolescents aged 8 to 18 years with congenital heart disease (mean age 13.4 ± 3.2 years, 53.3% female) who attended routine medical examinations at the German Heart Center Munich between May 2024 and August 2025. Information on the patient’s age, gender, height, weight, diagnosis, and treatment was collected from the medical records. In addition, 122 age- and sex-matched healthy controls (mean age 13.4 ± 3.1 years, 53.3% female) were recruited simultaneously from local schools in the Munich area. All study participants completed the Physical Activity Health Literacy Scale for Children (PAHL-C) and the Nutrition Health Literacy Scale for Children (NHL-C), and they self-reported the number of days they engaged in physical activity. Children were asked: “On how many days in an average week are you active for at least 60 min per day?” Responses ranged from 0 to 7 days, indicating the number of days per week the children were physically active for at least 60 min. This measure was used as a time-efficient way to assess weekly physical activity frequency.

Congenital heart defects were classified as mild, moderate, or complex according to the American College of Cardiology [15] and divided into six groups: ‘right heart obstruction’ (e.g., tetralogy of fallot, pulmonic stenosis); ‘left heart obstruction’ (e.g., aortic stenosis and coarctation of the aorta); ‘isolated shunts’ (ventricular, atrial, and atrioventricular septal defects); ‘transposition of the great arteries’; ‘univentricular hearts’; and ‘other’, which included all remaining structural cardiac abnormalities. In this outpatient clinic, all patients were classified as New York Heart Association class I or II. Exclusion criteria comprised the presence of genetic or chronic disorders, cognitive or psychomotor impairments, or any condition limiting the ability to complete or comprehend the questionnaires. Written consent was required from all study participants and their legal guardians. This study was conducted in accordance with the Declaration of Helsinki and the guidelines for good clinical practice and was approved by the Ethics Committee of the Technical University of Munich (project number: 314/14).

### 2.2. Physical Activity Health Literacy Scale for Children (PAHL-C)

The Physical Activity Health Literacy Scale for Children (PAHL-C) is an 8-item tool designed to assess children’s self-perceived health literacy regarding physical activity. It is part of a series of six survey instruments that measure health literacy across physical activity, nutrition, and psychosocial health [16]. The PAHL-C focuses on self-reported challenges in finding, understanding, evaluating, and using information related to healthy physical activity. Example questions from the questionnaire are: ‘How difficult or easy is it for you to assess whether you do enough physical activity and exercise each week to stay healthy and fit?’ and ‘How difficult or easy is it for you to choose physical activity and exercise instead of spending time watching TV or using your phone or computer?’. Responses are rated on a five-point Likert scale ranging from “very easy” to “very difficult.” The composite score, calculated on a scale from 0 to 100, reflects the level of physical activity-related health literacy, with higher scores indicating greater literacy. The scale demonstrates acceptable reliability, with a Cronbach’s alpha of 0.776 [17].

### 2.3. Nutrition Health Literacy Scale for Children (NHL-C)

The Nutrition Health Literacy Scale for Children (NHL-C) is an 8-item questionnaire developed to evaluate children’s self-perceived health literacy regarding nutrition [16]. The NHL-C assesses perceived difficulties in finding, understanding, evaluating, and applying information related to healthy eating. Example questions were: ‘How difficult or easy is it for you to understand the information on food packaging?’ and ‘How difficult or easy is it for you to choose fruit and vegetables instead of sweets or salty snacks?’. Responses are measured using a five-point Likert scale ranging from “very easy” to “very difficult.” The overall score, ranging from 0 to 100, indicates the level of health literacy related to nutrition, with higher scores reflecting greater literacy. The scale has demonstrated acceptable reliability, with a Cronbach’s alpha of 0.784 [18]. Although both instruments, the PAHL-C and the NHL-C, were originally tested with children aged 9 to 13, children from the age of 8 had already been included in the quantitative pretesting during the instrument development phase of the Geko-T project [16]. Therefore, in the present study, both questionnaires were used exploratively with 8-year-old participants. In cases of comprehension difficulties, support was provided by the study leader.

### 2.4. Data Analysis

Data analysis was performed using R (version 4.2.2) and R Studio (2024.09.1+394). Descriptive statistics were presented as mean values with standard deviations (mean ± SD) and absolute values and percentages if appropriate. The Shapiro–Wilk test was applied to assess normality of the data. Differences in PAHL-C and NHL-C between patients and controls were analyzed by comparing the mean scores using Student’s *t*-test. Health literacy scores and self-reported physical activity were compared across different types of congenital heart disease using a one-way ANOVA (aov() function). Normality of the residuals was assessed with the Shapiro–Wilk test, and homogeneity of variances was evaluated using Levene’s test. Non-parametric analyses were performed when necessary. In this case, the Kruskal–Wallis test was used, and post hoc pairwise comparisons were performed with the Wilcoxon rank-sum test. Statistical significance was defined as two-sided *p*-values below 0.05.

## 3. Results

**Figure 1:**
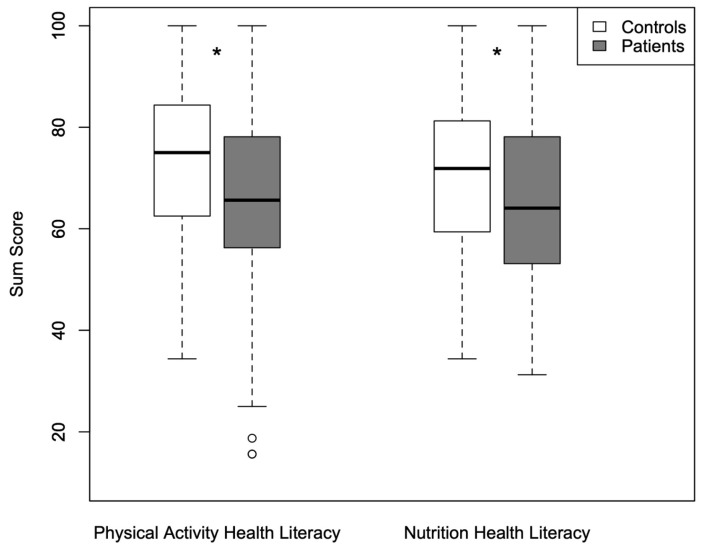
Physical activity health literacy and nutrition health literacy scores in children with congenital heart disease and control; boxplots showing the median (horizontal line), interquartile range (box), and minimum and maximum values (whiskers), with dots representing outliers; * significance level set at *p* < 0.05.

Table 1 provides a detailed overview of the characteristics of congenital heart disease patients and healthy controls. Significant differences were found between children and adolescents with congenital heart disease and healthy controls in PAHL-C scores (65.9 ± 18.0 vs. 72.9 ± 14.9, *p* = 0.001) and NHL-C scores (63.6 ± 19.0 vs. 69.3 ± 14.8, *p* = 0.009) as shown in Figure 1. Patients with congenital heart disease also reported less physical activity compared with controls (4.2 ± 1.7 vs. 5.2 ± 1.8 days/week, *p* < 0.001). No significant differences were found in terms of heart defect complexity in PAHL-C (simple: 68.3 ± 19.1; moderate: 68.6 ± 17.2; complex: 62.2 ± 18.1; *p* = 0.192), NHL-C (simple: 67.9 ± 15.6; moderate: 65.4 ± 16.7; complex: 60.5 ± 21.4; *p* = 0.539), or self-reported physical activity (simple: 3.9 ± 1.9; moderate: 4.2 ± 1.7; complex: 4.2 ± 1.8; *p* = 0.880).

In girls, PAHL-C scores were significantly lower in patients with congenital heart disease compared to healthy controls (Patients: 62.3 ± 16.6 vs. Control: 68.8 ± 15.6, *p* = 0.023). No significant differences were found in NHL-C scores (Patients: 63.9 ± 18.2 vs. Control: 66.4 ± 14.9, *p* = 0.393). Girls with congenital heart disease also reported less weekly physical activity (Patients: 3.6 ± 1.7 days/week vs. control: 4.9 ± 1.7 days/week, *p* < 0.001). In boys, both PAHL-C (Patients: 70.0 ± 18.8 vs. Control: 77.5 ± 12.6, *p* = 0.015) and NHL-C scores (Patients: 63.2 ± 20.0 vs. Control: 72.6 ± 14.1, *p* = 0.004) were significantly lower in patients. Similarly, they reported being significantly less active than the control group (Patients: 4.7 ± 1.7 vs. Control: 5.5 ± 1.7 days/week, *p* = 0.030).

## 4. Discussion

This study reveals that children and adolescents with congenital heart disease have lower health literacy in the areas of physical activity and nutrition and report less physical activity compared to their healthy peers. Neither the type of heart defect nor its complexity (simple, moderate, or complex) had an influence on health literacy scores. When analyzed by sex, girls with congenital heart disease demonstrated lower health literacy in the domains of physical activity and reported being less active than their healthy peers. Boys with congenital heart disease showed significantly lower health literacy in both physical activity and nutrition and reported being less active than the control group.

One possible explanation for the observed differences in health literacy and physical activity is that children with congenital heart disease often experience protective restrictions imposed by their social environment, including parents, teachers, and healthcare professionals. Avedissian et al. [19] reported that parents of children with congenital heart disease tend to display significantly higher levels of overprotective parenting and view their child as more vulnerable than parents of healthy children. Such protective behaviors may limit early learning opportunities for developing a positive and competence-enhancing relationship with physical activity and health-related decision-making. Repeated hospitalizations, performance comparisons with peers, or fear of overexertion may further weaken confidence in one’s physical abilities. Venna et al. [14] identified self-efficacy as the most important modifiable individual facilitator of physical activity; therefore, reduced self-efficacy may make it more difficult for children to not only understand but also translate knowledge about physical activity and nutrition into actual behavior.

Another explanation might be the strong medical focus on disease management rather than on health-promoting behaviors in the care of children and adolescents with congenital heart disease. Clinical care is predominantly oriented toward risk prevention, surgical interventions, regular medical check-ups, and symptom monitoring [20], while everyday health-related competencies such as autonomous physical activity planning or nutrition-related decision-making often receive less attention. As a result, health may primarily be experienced as something that is managed by the medical system and healthcare professionals, rather than as a modifiable and actively shapeable aspect of daily life. This disease-centered approach may limit opportunities for children and adolescents with congenital heart disease to develop health literacy skills that rely on autonomy, self-efficacy, and active engagement in health-related decision-making.

### 4.1. Health Literacy on Physical Activity and Nutrition

Research on health literacy among children with congenital heart disease is limited, particularly in the areas of physical activity and nutrition. Studies used other tools to assess health literacy, such as the Canadian Assessment of Physical Literacy (CAPL), which evaluates daily behavior (daily steps, self-reported active days per week), physical competence, motivation, and knowledge. These domains are assessed using questionnaires as well as children’s actual physical performance (e.g., shuttle run, plank, etc.). Longmuir et al. [21] used CAPL and reported that children aged 8 to 12 years with congenital heart disease did not differ from healthy peers in total physical literacy score, motivation, or knowledge. However, they showed significantly lower physical competence and accumulated more steps per day, while simultaneously reporting higher levels of sedentary time [21]. The authors noted that, besides the small sample size of patients with heart defects, the participants were predominantly individuals who were already more active, which may have led to a selection bias. A later analysis by Longmuir et al. [22] showed that no significant differences were observed in overall or subscale CAPL scores between children with congenital heart disease and healthy controls. In comparison, children with congenital heart disease in our study demonstrated lower health literacy related to physical activity and reported being less active, although daily steps were not assessed. These results are not directly comparable to CAPL-based studies due to methodological differences, as the CAPL also includes objective assessments of motor skills and fitness.

Studies using PAHL-C and NHL-C were conducted only on healthy children. Griebler et al. [16] examined a sample of 738 healthy boys and girls aged 9 to 13 years. In their study, boys showed significantly higher mean scores than girls in both the PAHL-C and the NHL-C domains. A similar pattern was observed in our control group, where boys also achieved higher scores in both areas, although overall scores were lower compared to those reported by Griebler et al. [16]. This difference may be explained by contextual factors such as differences between the Austrian and German school systems and their respective approaches to health education and physical activity promotion. Among children and adolescents with congenital heart disease, boys likewise demonstrated higher PAHL-C scores than girls in our study, while NHL-C scores were similar between sexes. Overall scores of congenital heart disease patients in our study are lower compared to those reported by Griebler et al. [16].

Research on health literacy in the domain of nutrition among children with chronic diseases, such as congenital heart disease, is still lacking. However, nutritional literacy may be particularly important in this population, as eating habits are critical determinants of health and strongly influence physical, emotional, and cognitive development [23]. Infants and children with congenital heart disease are especially prone to malnutrition, with reported prevalence rates ranging from 15% to 64% [24]. Adequate nutritional assessment and early-stage dietary interventions are therefore essential to support treatment success and improve clinical outcomes in this vulnerable group [25]. Beyond early childhood, healthy eating behaviors may continue to play a crucial role across the lifespan of children with congenital heart disease. In later life, appropriate dietary habits may help reduce the risk of developing obesity or type 2 diabetes, as well as other comorbidities. In addition, healthy nutrition may support optimal growth and development, enhance cognitive function, and promote better emotional well-being [26,27]. This is particularly relevant as the growing global obesity pandemic is increasingly affecting this population group and can become a major problem in children with congenital heart disease [28].

In contrast, research in healthy children suggests that they generally demonstrate a good understanding of basic nutritional concepts. A review by Bánfai-Csonka et al. [29] showed that healthy children are typically able to identify main food categories, distinguish between healthy and unhealthy foods, understand the relationship between portion size and health, and describe the health consequences of obesity.

Future research should therefore not only examine levels of nutritional health literacy in these children and adolescents with congenital heart disease, but also investigate effective strategies and interventions to promote age-appropriate and nutrition-related health literacy in this population.

### 4.2. Self-Reported Physical Activity

Noordstar et al. [30] examined 90 children aged 7 to 17 years with congenital heart disease who had undergone cardiac surgery within the first six months of life. In their study, children reported being physically active on an average of 5 days per week, which is higher than in our cohort (4.2 days/week). However, their sample included a different sex distribution (62 boys and 28 girls). Children with single ventricle physiology in their study reported being active on only 3 days per week, which is consistent with our findings. Longimur et al. [22] confirms our findings on self-reported activity behavior. Children with congenital heart disease reported fewer days of being physically active and more sedentary time compared to healthy peers, based on the number of days they achieved 12,000 steps. On average, children with congenital heart disease reached this target on 1.9 days per week, whereas healthy children did so on 5.3 days per week. While our study and previous findings indicate that children and adolescents with congenital heart disease exhibit lower health literacy and reduced self-reported physical activity compared to healthy peers, it is important to explore existing approaches to address these deficits. In this context, Barbazi et al. [31] did a scoping review about health educational interventions for children with congenital heart disease and found some studies that reported improvements in knowledge, self-management, coping strategies, empowerment, and quality of life, while others found no significant effects. The authors concluded that more targeted health education interventions are needed to support patients and improve their long-term health outcomes.

### 4.3. Limitations

This study did not assess socio-economic factors that may have influenced the results. Griebler et al. [16] demonstrated that factors such as school type, speaking a language other than German at home, and perceived household financial situation have a significant negative impact on PAHL-C and NHL-C scores. However, comparable data on children with congenital heart disease are not yet available. Moreover, self-reported physical activity may differ from objectively measured activity and may even overestimate actual activity levels, with participants reporting being active on more days than detected by accelerometer-based measurements [32]. Assessing objective physical activity parameters in addition to questionnaire-based measures would provide a more comprehensive picture of health literacy in this population.

## 5. Conclusions

Children and adolescents with congenital heart disease showed lower health literacy in both the physical activity and nutrition domains, as well as reduced self-reported physical activity compared to healthy controls. Only a few studies have examined health literacy in children and adolescents with congenital heart disease, and those that exist have used different assessment tools. The available evidence is therefore insufficient to determine the level of health literacy in this population, particularly in the domains of nutrition and physical activity. It underscores the need for further research in this area. Moreover, future studies should aim to identify determinants of health literacy and develop targeted interventions to promote health literacy from an early age. In the meantime, healthcare professionals, particularly pediatric cardiologists, should actively include physical activity and nutrition in discussions with patients and their families to support health-promoting behaviors.

## Figures and Tables

**Table 1:** Overview of children and adolescents with congenital heart disease classified by type of heart disease and healthy controls.

	*n* (%)	Sex, Female [*n* (%)]	Age, Years	BMI, kg/m^2^	Self-Reported Physical Activity, Days/Week	PAHL-C Score	NHL-C Score
Right Heart Obstruction	25 (20.5)	12 (18.5)	13.0 ± 2.6	18.9 ± 4.2	3.6 ± 1.6	62.4 ± 18.3	62.8 ± 13.8
Left Heart Obstruction	28 (23.0)	14 (21.5)	13.9 ± 3.4	20.2 ± 3.9	4.4 ± 1.7	72.5 ± 16.2	64.4 ± 22.4
Shunts	23 (18.9)	15 (23.1)	14.3 ± 3.5	20.2 ± 2.1	4.0 ± 1.7	68.1 ± 20.3	66.3 ± 18.4
TGA	15 (12.3)	6 (9.2)	13.6 ± 2.8	19.1 ± 3.3	4.9 ± 1.9	59.6 ± 20.2	61.2 ± 14.0
UVH	14 (11.5)	8 (12.3)	12.8 ± 2.8	18.6 ± 4.2	3.4 ± 1.8	62.7 ± 14.8	63.2 ± 15.2
Other	17 (13.9)	10 (15.4)	12.6 ± 3.8	17.0 ± 3.9	4.7 ± 1.5	63.8 ± 15.1	62.9 ± 27.9
Congenital heart disease	122	65 (53.3)	13.4 ± 3.1	19.1 ± 3.7	4.2 ± 1.7	65.9 ± 18.0	63.6 ± 19.0
Controls	122	65 (53.3)	13.4 ± 3.1	18.6 ± 2.9	5.2 ± 1.8	72.9 ± 14.9	69.3 ± 14.8
*p*-value *	-	-	0.988	0.193	<0.001	0.001	0.009

Mean ± standard deviation; TGA = transposition of the great arteries; UVH = univentricular heart; BMI = body mass index; Self-reported physical activity = number of days per week participants reported being active for at least 60 min; PAHL-C = Physical Activity Health Literacy Scale; NHL-C = Nutrition Health Literacy Scale for Children; * significance level set at *p* < 0.05.

## Data Availability

The data presented in this study are available on reasonable request from the corresponding author.

## Abbreviations

The following abbreviations are used in this manuscript:

WHO	World Health Organization
PAHL-C	Physical Activity Health Literacy Scale for Children
NHL-C	Nutrition Health Literacy Scale for Children
TGA	Transposition of the Great Arteries
UVH	Univentricular heart
